# MYC and BCL2 overexpression is associated with a higher class of Memorial Sloan-Kettering Cancer Center prognostic model and poor clinical outcome in primary diffuse large B-cell lymphoma of the central nervous system

**DOI:** 10.1186/s12885-016-2397-8

**Published:** 2016-06-10

**Authors:** Sehui Kim, Soo Jeong Nam, Dohee Kwon, Hannah Kim, Eunyoung Lee, Tae Min Kim, Dae Seog Heo, Sung Hye Park, Chul Woo Kim, Yoon Kyung Jeon

**Affiliations:** Department of Pathology, Seoul National University Hospital, Seoul National University College of Medicine, 101 Daehak-ro, Jongno-gu, Seoul, 03080 Republic of Korea; The Tumor Immunity Medical Research Center, Seoul National University College of Medicine, Seoul, Republic of Korea; Tumor Microenvironment Global Core Research Center, Seoul National University, Seoul, Republic of Korea; Department of Pathology, Asan Medical Center, Seoul, Republic of Korea; Department of Internal Medicine, Seoul National University Hospital, Seoul National University College of Medicine, Seoul, Republic of Korea

**Keywords:** Primary central nervous system lymphoma, Diffuse large B-cell lymphoma, MYC, BCL2, Prognosis

## Abstract

**Background:**

Primary diffuse large B-cell lymphoma of the central nervous system (PCNS-DLBCL) is a distinct clinicopathological entity with a poor prognosis. Concurrent MYC and BCL2 overexpression predicts inferior prognosis in systemic DLBCLs. However, the prognostic significance of MYC and BCL2 in PCNS-DLBCL remains elusive.

**Methods:**

Immunohistochemistry (IHC) of MYC, BCL2 and BCL6 was performed on tumor samples from 114 patients with PCNS-DLBCL. IHC score was assigned based on the proportion of immunostained cells.

**Results:**

MYC, BCL2, and BCL6 IHC scores were 18.16 ± 19.58, 58.86 ± 35.07, and 39.39 ± 37.66 % (mean ± SD), respectively. Twenty-one cases (18.1 %) were designated as MYC-positive with a cutoff score of 40. BCL2 positivity was found in 87 cases (75.0 %) using a cutoff score of 30. MSKCC (Memorial Sloan-Kettering Cancer Center prognostic model) class 2 and 3 had higher rates of MYC and/or BCL2 positivity (MYC, *P* = 0.012; BCL2, *P* = 0.008; dual-positive, *P* = 0.022). Poor KPS (Karnofsky Performance Status score <70), multifocal disease, Nottingham-Barcelona score ≥2, and MSKCC class 2 and 3 were related to shorter progression-free survival (PFS) (*P* = 0.001, 0.037, 0.001, and 0.008, respectively). Patients with older age (>60 years) showed poorer overall survival (OS) (*P* = 0.020). MYC positivity was associated with poor PFS (*P* = 0.027), while patients with BCL2 positivity exhibited a shorter OS (*P* = 0.010). Concomitant MYC and BCL2 positivity was related to poor PFS (*P* = 0.041), while the lack of both MYC and BCL2 expression was related to prolonged OS (*P* = 0.014). MYC and BCL2 expression had no independent prognostic implication by multivariate analysis in overall patients with PCNS-DLBCL. However, among patients treated with combined high-dose methotrexate, vincristine and procarbazine and radiotherapy, dual MYC and BCL2 overexpression (a cutoff score of 60) was an independent poor prognostic indicator (*P* = 0.010).

**Conclusions:**

Evaluation of MYC and BCL2 expression may be helpful for the determination of PCNS-DLBCL prognosis.

**Electronic supplementary material:**

The online version of this article (doi:10.1186/s12885-016-2397-8) contains supplementary material, which is available to authorized users.

## Background

Primary diffuse large B-cell lymphoma of the central nervous system (PCNS-DLBCL) is a distinct subtype of DLBCL that primarily arises in the intracranial and intraocular areas [1]. PCNS-DLBCL has a poorer prognosis than systemic DLBCL. This may be due to the fact the blood brain barrier makes it difficult for chemotherapeutic agents to penetrate, that the site of involvement is immune-privileged, and that the cell of origin is mainly a non-germinal center B-cell [2].

To determine the prognosis of PCNS-DLBCL, several scoring systems, which are mostly based on clinical features, have been recommended. The international extranodal lymphoma study group (IELSG) suggested that age (>60 years), performance status (PS) (≥2), elevated serum lactate dehydrogenase (LDH) levels, a high cerebrospinal fluid (CSF) protein concentration, and involvement of deep brain structures (periventricular regions, basal ganglia, brain stem, and/or cerebellum) are significantly associated with a poor prognosis. This scoring system was validated in patients treated with high-dose methotrexate-based chemotherapy [3]. In contrast, the Nottingham/Barcelona score, which incorporates age ≥60, PS ≥ 2 and multifocal disease, showed a prognostic impact in patients treated with CHOD/BVAM or BVAM chemotherapy followed by radiotherapy [4]. Meanwhile, the Memorial Sloan-Kettering Cancer Center (MSKCC) prognostic model is able to classify patients into class 1 (age ≤50 years), class 2 (age >50; Karnofsky performance score (KPS) ≥70) and class 3 (age >50; KPS <70) with prognostic significance [5]. However, there is still no consensus about prognostic scoring systems in PCNS-DLBCL. Moreover, the pathological and biological prognostic factors for PCNS-DLBCL remain unknown.

Recent studies have shown that concomitant MYC and BCL2 expression predicts inferior survival in systemic DLBCL patients treated with rituximab-CHOP [6, 7]. Moreover, coexpression MYC and BCL2 was found to have prognostic value in patients with DLBCL independent of cell of origin, but is related to a high-risk gene expression signature [7]. Thus, MYC and BCL2 coexpression status is very helpful for risk stratification along with the international prognostic index (IPI) score in systemic DLBCL [6, 7]. Meanwhile, only a few studies are available on MYC and BCL2 expression in PCNS-DLBCL [8–10]. However, these studies have demonstrated conflicting results in terms of prognostic significance of MYC and BCL2 status in patients with PCNS-DLBCL. A study on 47 patients with PCNS-DLBCL showed that MYC, BCL2 and BCL6 were frequently coexpressed but had no prognostic significance [8]. Another study demonstrated that MYC expression, with or without BCL2 coexpression, was not predictive of clinical outcome in 59 patients with PCNS-DLBCL [9]. In contrast, MYC expression was associated with poor prognosis in a study on 42 patients with PCNS-DLBCL [10]. Thus, we comprehensively investigated the expression of MYC, BCL2 and BCL6, and their association with clinicopathological characteristics and prognosis in a large cohort of PCNS-DLBCL patients.

## Methods

### Patients

In total, 114 patients diagnosed with PCNS-DLBCL managed at Seoul National University Hospital (SNUH, Seoul, Korea) between 2000 and 2012 were included in this study. Pathologic diagnosis was performed according to the current World Health Organization (WHO) classification of Tumors of Hematopoietic and Lymphoid Tissues [1]. Tumor tissues were obtained before treatment in all patients. Clinicopathological and survival data were retrieved from medical records by three hemato-oncologists (E.L, T.M.K and D.S.H) and from the review of pathological material by three pathologists (S.K., S.J.N and Y.K.J). This study followed the World Medical Association Declaration of Helsinki recommendations and was approved by the Institutional Review Board (IRB) of SNUH (IRB No. 1506-080-681). Informed consent for participation in the study was waived by the IRB of SNUH on the basis that this study was a retrospective study using archived material and did not pose increased risk to the patients.

### Immunohistochemistry

Immunohistochemistry (IHC) was performed using antibodies against MYC (clone EP121, Cell Marque, Rocklin, CA, USA), BCL2 (clone 124, DAKO, Carpinteria, CA, USA), BCL6 (clone LN22, Novocastra, Newcastle, United Kingdom), CD10 (clone 56C6, Novocastra), and MUM1 (clone Ma695, Novocastra). IHC staining was performed using the Ventana Benchmark XT system (Ventana Medical Systems, Tucson, AZ, USA) or a Bond-Max automated immunostainer (Leica Microsystems, Melbourne, Australia). Cell of origin was assessed according to the Hans criteria [11]. IHC score was determined to be the percentage of tumor cells with robust immunostaining evaluated by 10 % increments. Cutoff values (i.e. IHC scores) of ≥40 % for MYC, ≥30 and ≥60 % for BCL2 and ≥50 % for BCL6 were determined to have discriminant prognostic power based on the receiver operator characteristic (ROC) curves and were thus used for classifying cases into MYC-, BCL2- or BCL6-negative and-positive groups.

### Statistical analysis

A comparison of clinicopathological parameters was performed by Chi-square, Fisher’s exact, and Student’s *t*-tests. PFS and OS were analyzed using the Kaplan-Meier method and the log-rank test. Univariate and multivariate analysis were performed using the Cox proportional hazards regression model. All statistical analyses were performed using SPSS software (version 21; IBM Corp., Armonk, NY, USA). Two-sided *P* values <0.05 were considered statistically significant.

## Results

### Clinicopathological features of patients with PCNS-DLBCL

The clinicopathological characteristics and treatment modalities of 114 patients with PCNS-DLBCL are summarized in Table 1. The patient population included 65 (57.0 %) males and 49 (43.0 %) females with a median age of 61 years (range 10–82 years). Most patients had experienced neurologic deficits as the initial symptom (78/114, 68.4 %), and had a KPS score ≥70 (97/111, 87.4 %) and an ECOG PS <2 (73/112, 65.2 %) at diagnosis. Deep structure involvement (84/114, 73.7 %) and multifocality (72/114, 63.2 %) were frequently observed. The majority (89/113, 78.8 %) of cases were of non-germinal center B-cell origin, which was higher than in systemic DLBCL patients in our institute (63 %) [12]. In total, 52.7 % (48/91) of patients had an IELSG score ≥ 3, 52.7 % (59/112) of patients had a Nottingham-Barcelona score ≥2 and 79.8 % (91/112) of patients were classified as MSKCC class 2 or 3. Most patients received high-dose methotrexate-containing chemotherapy including high-dose methotrexate, vincristine and procarbazine (MVP) (79/97, 81.4 %) or high-dose methotrexate (HD-MTX) (14/97, 14.4 %), and 58.8 % (57/97) of patients were treated with combined MVP and radiotherapy.

**Table 1:** Clinicopathological characteristics of patients and treatment modalities

Variables^a^		n (%)	5YRS	PFS, *P* ^d^	5YRS	OS, *P* ^d^
		(Total = 114)	PFS (SD)		OS (SD)	
Age (yr)	≤50	23 (20.2)	0.684 (0.108)	0.072	0.909 (0.043)	0.126
	>50	91 (79.8)	0.334 (0.090)		0.698 (0.086)	
Age (yr)	≤60	55 (48.2)	0.498 (0.081)	0.585	0.854 (0.065)	0.014
	>60	59 (51.8)	0.217 (0.167)		0.569 (0.172)	
Sex	M	65 (57.0)	0.452 (0.086)	0.708	0.849 (0.054)	0.156
	F	49 (43.0)	0.460 (0.112)		0.452 (0.168)	
Initial Symptoms	Headache &vomiting	30 (26.3)	0.609 (0.114)	0.400	0.870 (0.071)	0.266
	Seizure	6 (5.3)	0.000 (0.000)		100.0	
	Neurologic deficit	78 (68.4)	0.387 (0.085)		0.697 (0.085)	
ECOG PS	0, 1	73 (65.2)	0.425 (0.086)	0.818	0.789 (0.075)	0.247
	2–4	39 (34.8)	0.511 (0.135)		0.752 (0.079)	
KPS	≥70	97 (87.4)	0.468 (0.082)	0.001	0.760 (0.065)	0.854
	<70	14 (12.6)	0.259 (0.144)		0.821 (0.117)	
B Symptoms	Absent	109 (95.6)	0.452 (0.073)	0.284	0.768 (0.061)	0.558
	Present	5 (4.4)	0.333 (0.272)		0.800 (0.179)	
Serum LDH	Normal	68 (63.6)	0.223 (0.087)	0.005	0.767 (0.102)	0.201
	Elevated	39 (36.4)	0.675 (0.102)		0.760 (0.100)	
Cell of origin	GCB	24 (21.2)	0.689 (0.120)	0.073	0.795 (0.092)	0.957
	Non-GCB	89 (78.8)	0.383 (0.082)		0.756 (0.074)	
Involvement of deep structure	Absent	30 (26.3)	0.441 (0.161)	0.219	0.758 (0.129)	0.640
	Present	84 (73.7)	0.433 (0.078)		0.762 (0.070)	
Extent of disease	Unifocal	42 (36.8)	0.553 (0.119)	0.037	0.753 (0.137)	0.603
	Multifocal	72 (63.2)	0.368 (0.088)		0.782 (0.062)	
Ocular involvement	Absent	101 (89.4)	0.485 (0.072)	0.985	0.872 (0.046)	0.466
	Present	12 (10.6)	0.233 (0.190)		0.525 (0.204)	
CSF protein	Normal	39 (42.4)	0.291 (0.142)	0.988	0.675 (0.141)	0.340
	Elevated	53 (57.6)	0.467 (0.090)		0.849 (0.058)	
CSF cytology	Negative	82 (85.4)	0.356 (0.083)	0.281	0.774 (0.066)	0.693
	Positive	14 (14.6)	0.608 (0.158)		0.711 (0.180)	
IELSG	0–2	43 (47.3)	0.229 (0.100)	0.093	0.853 (0.101)	0.054
	3–5	48 (52.7)	0.638 (0.088)		0.736 (0.074)	
Nottingham– Barcelona	0–1	53 (47.3)	0.604 (0.097)	0.001	0.807 (0.080)	0.188
	2–3	59 (52.7)	0.264 (0.098)		0.723 (0.084)	
MSKCC class	1	21 (18.4)	0.760 (0.106)	0.008	0.847 (0.108)	0.157
	2–3	91 (79.8)	0.340 (0.086)		0.688 (0.095)	
Radiotherapy	Not done	36 (31.6)	0.586 (0.142)	0.854	0.758 (0.086)	0.057
	Done	78 (68.4)	0.416 (0.080)		0.787 (0.068)	
Chemotherapy	MVP^b^	79 (81.4)	0.431 (0.084)	0.519	0.772 (0.072)	0.940
	HD-MTX	14 (14.4)	0.442 (0.176)		0.791 (0.138)	
	Others^c^	4 (4.1)	0.750 (0.217)		100.0	
Rituximab	Not done	103 (90.4)	0.453 (0.074)	0.756	0.748 (0.067)	0.503
	Done	11 (9.6)	0.333 (0.248)		0.909 (0.087)	
IT-MTX	Not done	89 (78.1)	0.412 (0.092)	0.649	0.743 (0.076)	0.415
	Done	25 (21.9)	0.457 (0.114)		0.832 (0.089)	

*Abbreviations*: *ECOG PS* The Eastern Cooperative Oncology Group performance score, *KPS* Karnofsky performance status score, *LDH* lactate dehydrogenase, *GCB* germinal center B cell-like, *CSF* cerebrospinal fluid, *IELSG* the International Extranodal Lymphoma Study group, *MSKCC* Memorial Sloan Kettering Cancer Center, *MVP* combined chemotherapy regimen of high-dose methotrexate, vincristine and procarbazine, *HD-MTX* high-dose methotrexate, *IT-MTX* intrathecal methotrexate, *PFS* progression-free survival, *OS* overall survival

^a^Some cases have missing values that lacked the information about the variables

^b^Includes 8 patients who received rituximab-MVP

^c^Others include COPADM and CHOP

^d^PFS and OS were analyzed using the Kaplan-Meier method with the log-rank test

The five-year PFS and OS based on clinicopathological variables are shown in Table 1. Older patients (age >60) had a shorter OS (*P* = 0.014), and patients with KSP <70 and multifocal lesions had a shorter PFS (*P* = 0.001 and 0.037, respectively). The non-germinal center phenotype tended to have a poorer PFS, but this did not reach statistical significance (*P* = 0.073). Patients with a Nottingham-Barcelona score ≥2 or MSKCC class 2 or 3 had a shorter PFS (*P* = 0.001 and 0.008, respectively). The IELSG scoring system showed inconclusive results, in that patients with an IELSG score ≥3 had a tendency to show better PFS (*P* = 0.093) and worse OS (*P* = 0.054). The treatment modalities including the chemotherapeutic agent, use of radiotherapy, rituximab or intrathecal-MTX were not associated with patient clinical outcomes in the total patient group. However, in the MVP-treated group, patients receiving radiotherapy showed a better prognosis than did those who did not receive radiotherapy (for PFS, *P* = 0.078; for OS, *P* = 0.001) (data not shown).

### Relationship between MYC, BCL2, and BCL6 expression and clinicopathological features

**Fig. 1:**
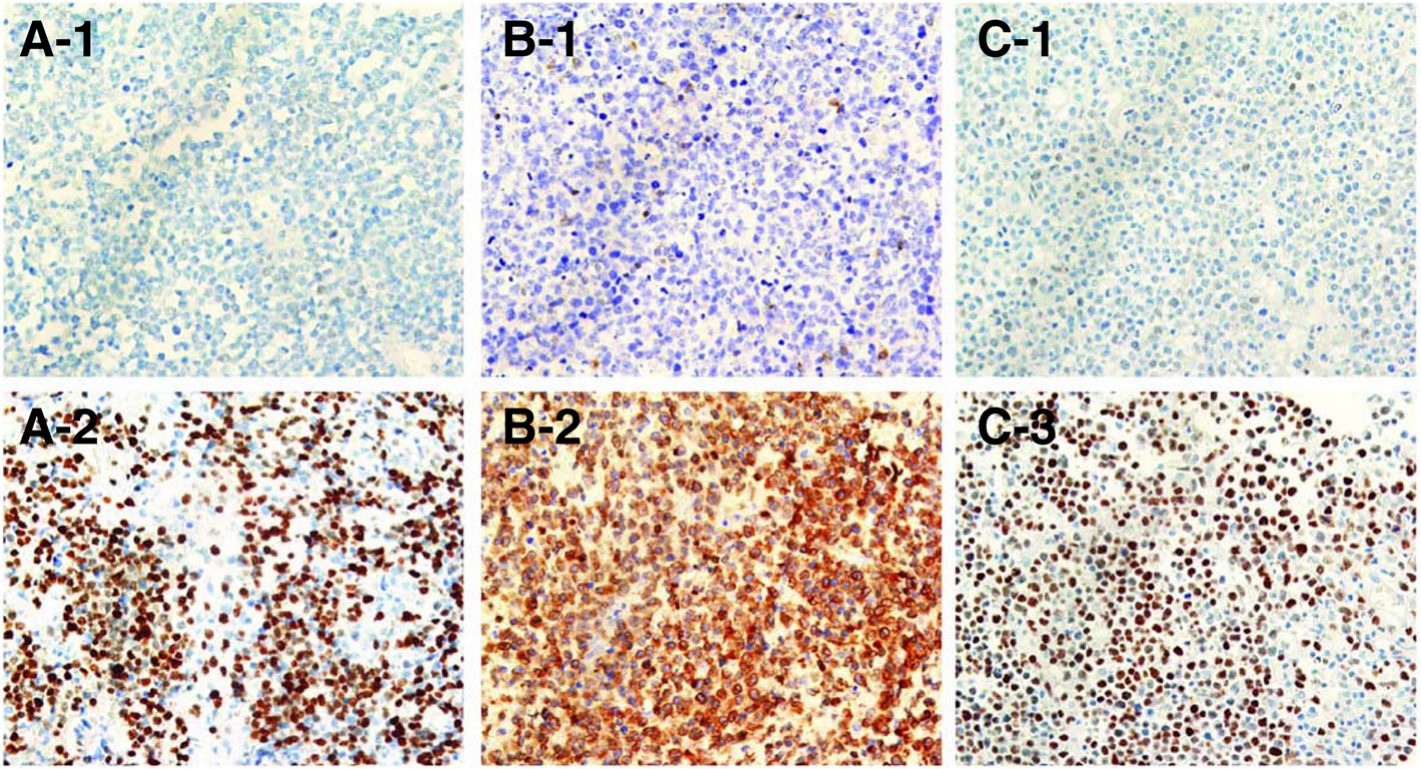
Representative immunohistochemical images of MYC, BCL2 and BCL6 expression in PCNS-DLBCL tumors. Representative IHC images of MYC-negative (**A**-**1**) and **-**positive (**A**-**2**) cases, BCL2-negative (**B**-**1**) and -positive (**B**-**2**) cases, and BCL6-negative (**C**-**1**) and -positive (**C**-**2**) cases

Representative IHC images of MYC, BCL2 and BCL6 are shown in Fig. 1. The proportion of tumor cells expressing MYC (i.e., MYC IHC score) was estimated as 18.16 ± 19.58 % (mean ± SD) (median: 10, range: 0-80 %). Using a cutoff score of 40, 21 (18.1 %) of the 114 cases were placed in the MYC-positive group. The BCL2 IHC score was also measured, with an average score of 58.86 ± 35.07 % (median: 70, range: 0-100 %); 87 (75.0 %) and 68 (59.6 %) of the 114 cases were classified as BCL2-positive using cutoff score of 30 and 60, respectively. The average BCL6 IHC score was 39.39 ± 37.66 % (median: 25, range: 0–100 %), and 51 (44.0 %) of the 114 cases were classified as BCL6-positive using a cutoff score of 50. Taken together, MYC and BCL2 dual-positive cases accounted for 15.8 % (18/114) of PCNS-DLBCLs, while MYC and BCL2 dual-negative cases accounted for 21.9 % (25/114) with a BCL2 cutoff score of 30. In addition, MYC and BCL2 IHC scores were positively correlated (Spearman’s rho = 0.320, *P* = 0.001), but MYC and BCL6, and BCL2 and BCL6 IHC scores were not (Additional file 1: Figure S1).

Among the 57 patients treated with combined MVP and radiotherapy (MVP-RT hereafter), 12.3 % (7/57) were designated as MYC-positive using a cutoff score of 40, and 71.9 % (41/57) and 54.4 % (31/57) of the cases were classified as BCL2-positive using cutoff scores of 30 and 60, respectively; 49.1 % (28/57) of the cases were classified as BCL6-positive using a cutoff score of 50. Taken together, MYC and BCL2 dual-positive cases accounted for 12.3 % (7/57; with a BCL2 cutoff score of 30) and 10.5 % (6/57; with a BCL2 cutoff score of 60), while MYC and BCL2 dual-negative cases accounted for 28.1 % (16/57; with a BCL2 cutoff score of 30) and 43.9 % (25/57; with a BCL2 cutoff score of 60) of patients in the MVP-RT group.

The relationship between MYC, BCL2 and BCL6 expression and clinicopathological features are summarized in Table 2 and Additional file 2: Table S1. Patients aged > 60 years tended to be BCL2-positive more often (*P* = 0.051), and higher MSKCC class was significantly associated with MYC and BCL2 positivity separately (*P* = 0.012 and 0.008, respectively) (Table 2) and with MYC and BCL2 coexpression (*P* = 0.022 by Fisher’s Exact Test) (data not shown). Cases of non-germinal cell origin were BCL2-positive more often (*P* = 0.005), but were BCL6-negative (*P* = 0.013) (Table 2 and Additional file 2: Table S1). There were no other correlations between clinical parameters and MYC, BCL2 or BCL6 expression. In addition, MYC, BCL2 or BCL6 positivity did not differ by treatment modality (data not shown). Among the patients of MVP-RT group, no significant relationship was observed between clinicopathological features and the MYC and BCL2 expression status except for higher expression of MYC in female than in male (*P* = 0.039) (data not shown).

**Table 2:** Correlation of MYC and BCL2 expression and clinicopathological variables

		MYC, n (%)		BCL2, n (%)			
Variables^a^		<40	≥40	*P*	<30	≥30	*P*
Age (yr)	mean ± SD	57.6 ± 14.6	63.1 ± 7.6	0.097	50.4 ± 19	61.3 ± 10.4	<0.001
Age (yr)	≤50	22 (95.7)	1 (4.3)	0.069	11 (47.8)	12 (52.2)	0.004
	>50	71 (78)	20 (22)		17 (18.7)	74 (81.3)	
Age (yr)	≤60	46 (83.6)	9 (16.4)	0.584	18 (32.7)	37 (67.3)	0.051
	>60	47 (79.7)	12 (20.3)		10 (16.9)	49 (83.1)	
Sex	M	55 (84.6)	10 (15.4)	0.335	20 (30.8)	45 (69.2)	0.076
	F	38 (77.6)	11 (22.4)		8 (16.3)	41 (83.7)	
ECOG PS	0, 1	62 (84.9)	11 (15.1)	0.172	19 (26)	54 (74)	0.731
	2–4	29 (74.4)	10 (25.6)		9 (23.1)	30 (76.9)	
KPS	≥70	81 (83.5)	16 (16.5)	0.136	24 (24.7)	73 (75.3)	>0.999
	<70	9 (64.3)	5 (35.7)		3 (21.4)	11 (78.6)	
B symptoms	Absent	88 (80.7)	21 (19.3)	0.582^b^	26 (23.9)	83 (76.1)	0.595^b^
	Present	5 (100)	0 (0)		2 (40)	3 (60)	
Serum LDH	Normal	54 (79.4)	14 (20.6)	0.506	19 (27.9)	49 (72.1)	0.582
	Elevated	33 (84.6)	6 (15.4)		9 (23.1)	30 (76.9)	
Cell of origin	GCB	22 (91.7)	2 (8.3)	0.236^b^	11 (45.8)	13 (54.2)	0.005
	Non-GCB	70 (78.7)	19 (21.3)		16 (18)	73 (82)	
Involvement of deep structure	Absent	24 (80)	6 (20)	0.795	6 (20)	24 (80)	0.499
	Present	69 (82.1)	15 (17.9)		22 (26.2)	62 (73.8)	
Extent of disease	Unifocal	34 (81)	8 (19)	0.895	11 (26.2)	31 (73.8)	0.758
	Multifocal	59 (81.9)	13 (18.1)		17 (23.6)	55 (76.4)	
Ocular involvement	Absent	82 (81.2)	19 (18.8)	>0.999^b^	25 (24.8)	76 (75.2)	>0.999^b^
	Present	10 (83.3)	2 (16.7)		3 (25)	9 (75)	
CSF protein	Normal	31 (79.5)	8 (20.5)	0.844	8 (20.5)	31 (79.5)	0.157
	Elevated	43 (81.1)	10 (18.9)		18 (34.0)	35 (66.0)	
CSF cytology	Negative	66 (80.5)	16 (19.5)	>0.999^b^	19 (23.2)	63 (76.8)	0.121
	Positive	11 (78.6)	3 (21.4)		6 (42.9)	8 (57.1)	
IELSG	0–2	35 (81.4)	8 (18.6)	0.790	13 (30.2)	30 (69.8)	0.740
	3–5	38 (79.2)	10 (20.8)		13 (27.1)	35 (72.9)	
Nottingham- Barcelona	0–1	46 (86.8)	7(13.2)	0.154	16 (30.2)	37 (69.8)	0.229
	2–3	45 (76.3)	14 (23.7)		12 (20.3)	47(79.7)	
MSKCC class	1	21 (100)	0 (0)	0.012^b^	10 (47.6)	11 (52.4)	0.008
	2–3	70 (76.9)	21 (23.1)		18 (19.8)	73 (80.2)	

*Abbreviations*: *ECOG PS* The Eastern Cooperative Oncology Group performance score, *KPS* Karnofsky performance status score, *LDH* lactate dehydrogenase, *GCB* germinal center B cell-like, *CSF* cerebrospinal fluid, *IELSG* the International Extranodal Lymphoma Study group, *MSKCC* Memorial Sloan Kettering Cancer Center

^a^Some cases have missing values that lacked the information about the variables

^b^Fisher’s exact test

### Prognostic significance of MYC, BCL2 and BCL6 expression

**Fig. 2:**
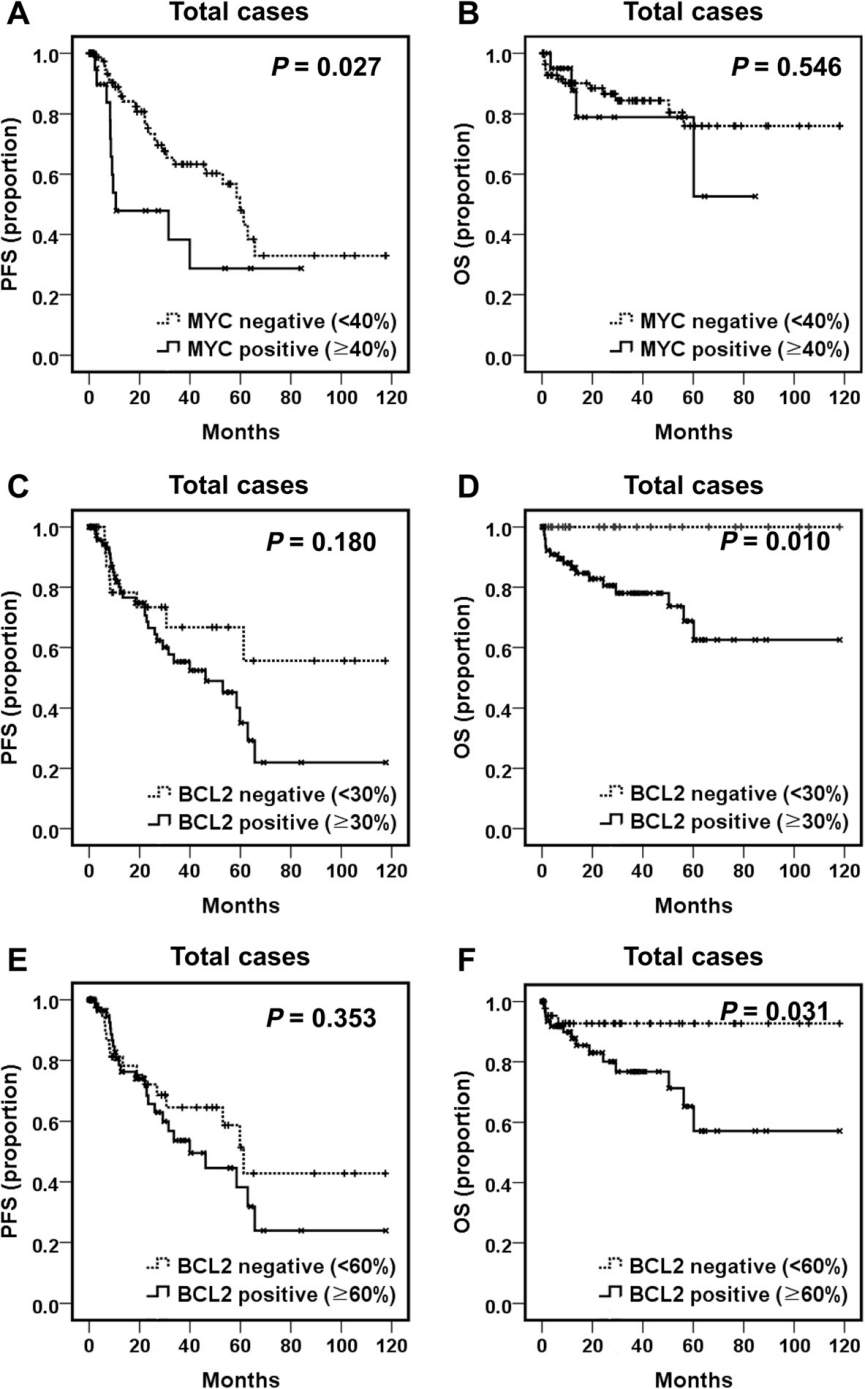
Progression-free survival (PFS) and overall survival (OS) of patients with PCNS-DLBCL according to MYC or BCL2 expression status. **a** and **b** PFS and OS according to MYC protein expression status (cutoff score 40) are plotted using the Kaplan-Meier method and analyzed by the log-rank test. PFS and OS according to BCL2 protein expression status using a cutoff score of 30 (**c** and **d**), or with a cutoff score of 60 (**e** and **f**) are plotted using the Kaplan-Meier method and analyzed by the log-rank test

**Fig. 3:**
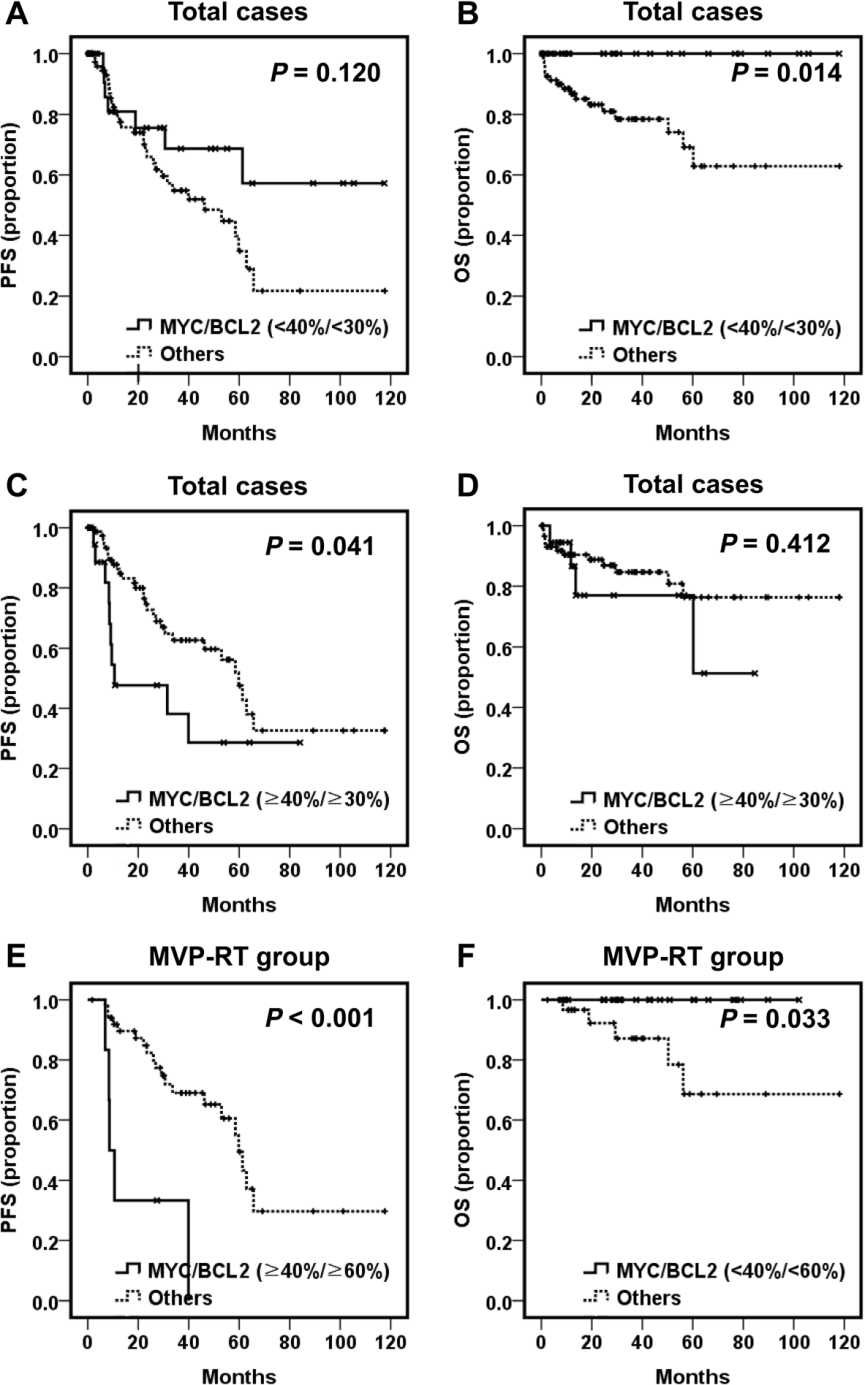
Progression-free survival (PFS) and overall survival (OS) of patients with PCNS-DLBCL according to combined MYC and BCL2 expression status. **a** and **b** PFS and OS of patients without expression for either MYC or BCL2 (double-negative) versus other groups are plotted using the Kaplan-Meier method and analyzed by the log-rank test. **c** and **d** PFS and OS of patients with concomitant MYC and BCL2 expression (double-positive) versus other groups are plotted using the Kaplan-Meier method and analyzed by the log-rank test. **e** and **f** PFS and OS of patients treated with combined MVP and radiotherapy according to the MYC and BCL2 expression status were plotted using the Kaplan-Meier method and analyzed by the log-rank test

Kaplan-Meier survival analysis demonstrated that MYC expression (cutoff score 40) was significantly associated with a shorter PFS (*P* = 0.027) in patients with PCNS-DLBCL (Fig. 2a), but not with OS (Fig. 2b). In contrast, higher BCL2 expression was significantly related to a shorter OS (*P* = 0.010 with a cutoff score of 30 and *P* = 0.031 with a cutoff score 60), but was not related to PFS (Fig. 2c-f). BCL6 expression had no prognostic significance. Patients who were negative for both MYC and BCL2 (cutoff score 30) showed better OS than patients positive for either (*P* = 0.014) (Fig. 3a and b). Patients with dual positivity for MYC and BCL2 (cutoff score 30) exhibited worse PFS than that of the others (*P* = 0.041) (Fig. 3c and d), but its statistical significance was lower than that observed by comparing MYC expression status alone (Fig. 2a). After incorporating both age and prognostic score into a multivariate analysis, MYC and BCL2 expression lost its prognostic impact, and a higher Nottingham-Barcelona score (≥2) and age remained as independent prognostic indicators for PFS (*P* = 0.032) and OS (*P* = 0.044), respectively (Table 3).

**Table 3:** Multivariate analysis of MYC expression and prognostic scoring systems for progression-free survival and overall survival in all patients with PCNS-DLBCL

	PFS			OS			
	HR	95 % CI	*P*		HR	95 % CI	*P*
Age	0.989	0.949-1.032	0.614	Age	1.074	1.002–1.151	0.044
MYC ≥ 40	1.745	0.848–3.589	0.130	BCL2 ≥ 30	-^a^	-^a^	0.951
N-B, 2-3	2.243	1.072–4.693	0.032	N-B, 2-3	1.392	0.474–4.084	0.547
MSKCC, 2–3	2.635	0.502–13.827	0.252	MSKCC, 2–3	0.370	0.048–2.841	0.339

*Abbreviations*: *PFS* progression-free survival, *OS* overall survival, *HR* hazard ratio, *CI* confidence interval, *N-B* Nottingham-Barcelona, *MSKCC* Memorial Sloan Kettering Cancer Center

(^a^values are not shown.)

Among patients with PCNS-DLBCL treated with MVP-RT, MYC expression (cutoff score of 40) was associated with a shorter PFS (*P* = 0.011) (Additional file 3: Figure S2). BCL2 overexpression (cutoff score of 60) was significantly related to a shorter OS (*P* = 0.025) (Additional file 3: Figure S2). In the MVP-RT group, patients with dual positivity for MYC and BCL2 (cutoff score of 60) exhibited worse PFS than that of the others (*P* <0.001) (Fig. 3e), and those who were negative for both MYC and BCL2 (cutoff score of 60) showed better OS than that of patients positive for either (*P* = 0.033) (Fig. 3f). Furthermore, in multivariate analysis, MYC and BCL2 coexpression was an independent factor for poor PFS (*P* = 0.010; hazard ratio = 4.372) (Table 4).

**Table 4:** Multivariate analysis of MYC and BCL2 coexpression and prognostic scoring systems for progression-free survival in patients with PCNS-DLBCL treated with combined MVP and radiotherapy

	PFS		
	HR	95 % CI	*P*
Age	0.983	0.922–1.049	0.605
N-B, 2–3	1.146	0.464–2.830	0.768
MSKCC, 2–3	4.975	0.715–34.613	0.105
MYC (≥40) and BCL2 (≥60), dual positive	4.372	1.430–13.367	0.010

*Abbreviations*: *PFS* progression-free survival, *HR* hazard ratio, *CI* confidence interval, *N-B* Nottingham- Barcelona, *MSKCC* Memorial Sloan Kettering Cancer Center

## Discussion

This study demonstrated that MYC and BCL2 overexpression was significantly associated with a high MSKCC class, and was related to shorter PFS and OS in patients with PCNS-DLBCL.

This study showed that MYC overexpression was associated with poor PFS in PCNS-DLBCL, which was partly consistent with Tapia et al.’s study [10], but different from previous reports by Brunn et al. and Gill et al. [8, 9]. Tapia et al. reported that MYC overexpression (using a cutoff score of 30) was related to poor OS, while the latter studies showed that MYC expression was not associated with PCNS-DLBCL prognosis. In this study, the mean MYC expression score was 18.16 ± 19.58 and the MYC positive rate using a cutoff score of 40 was 18.1 %, which were much lower than those observed in previous reports. Previous studies on PCNS-DLBCL reported a mean MYC expression score of 29–50 and MYC positive rates ranging from 43 to 82 % with cutoff values of 30 or 40, and an unreported cutoff score in one case [8–10]. However, antibody and racial differences (Western *vs.* Asian) may account for these discrepancies.

MYC immunostaining in systemic DLBCLs, particularly in cases without *MYC* gene translocation, is heterogeneous. Thus, the feasibility of interpreting and scoring MYC expression using IHC in DLBCL has been questioned [13]. We performed *MYC* fluorescence in situ hybridization in PCNS-DLBCL cases with MYC overexpression as reported previously [14]. Of note, in this study, only 2 (12 %) of 17 patients with MYC overexpression had a *MYC* translocation, and another two patients showed increased *MYC* copy number (ICN) (Additional file 2: Table S2). In contrast, approximately 25 % of the MYC overexpressing systemic DLBCLs showed *MYC* gene translocation [15]. Thus, translocation and ICN did not appear to explain MYC overexpression in most cases of PCNS-DLBCL, consistent with a previous report [10]. MYC overexpression in PCNS-DLBCL might result from other mechanisms such as a mutation of *MYC* and post-transcriptional or post-translational regulation. In addition, post-genetic or epigenetic regulation of MYC expression in PCNS-DLBCL may lead to heterogeneous MYC immunostaining. Meanwhile, concordance of MYC scoring between hematopathologists was much lower when interpreting entire tissue sections rather than a tissue microarray using a 1-mm core [13]. In this study, whole tissue sections were used and the largest series of patients with PCNS-DLBCL was evaluated based on treatment modality, and MYC overexpression was found to have prognostic value.

The rate of BCL2 expression in PCNS-DLBCL varies, which might also be attributable to the use of different antibodies and different cutoff values for determining overexpression [8, 9, 16–19]. Previous studies reported a wide range of BCL2 expression rates (56–92 %) with various cutoff scores from 25–70. We observed that 75.0 % (87/114) of PCNS-DLBCL cases were BCL2-positive using a cutoff score of 30, similar to a previous report by Tapia et al. [10]. In their study, BCL2 positivity was observed in 71 % of PCNS-DLBCL cases, but had no relationship with prognosis [10]. In contrast, the present study demonstrated that patients with PCNS-DLBCL and BCL2 overexpression tended to have a shorter PFS and had significantly poorer OS, suggesting that BCL2 may potentially be used as a prognostic marker.

In this study, MYC and BCL2 expression lost their prognostic significance after multivariate analysis. This may be partly attributable to the fact that MYC and BCL2 expression was significantly associated with higher MSKCC class. The MYC and BCL2 coexpression rate was 15.8 % (18/114) of PCNS-DLBCLs, which was much lower than values from previous studies including 29 % (12/41), 60 % (35/59) and 82 % (41/50) of PCNS-DLBCLs and 34 % (157/466) of systemic DLBCLs [7–10], but was similar to the rate (21 % [64/304]) reported in another study on systemic DLBCLs [6]. In this study, patients with PCNS-DLBCL and concomitant MYC and BCL2 overexpression showed poor PFS (*P* = 0.041), and those lacking both MYC and BCL2 overexpression had a prolonged OS (*P* = 0.014). However, the statistical significance of MYC and BCL2 dual-positivity on the PFS of patients was diminished compared to MYC positivity alone (*P* = 0.027). Similar to the present study, Tapia et al. reported that high MYC expression was associated with a lower OS, but that concurrent expression of MYC and BCL2 showed a tendency towards a lower OS with no statistical significance [10]. However, in this study, when analyzed in patients with PCNS-DLBCL treated with combined MVP and radiotherapy, MYC and BCL2 dual-positivity was an independent prognostic factor for poor PFS. Thus, MYC and BCL2 coexpression in PCNS-DLBCL seems to have prognostic value, although it is limited compared with systemic DLBCL.

Several previous studies are available on BCL6 expression and its prognostic impact on PCNS-DLBCL. BCL6 expression rates from 46 to 79 % of PCNS-DLBCL cases with cutoff scores from 10–60 have been reported [16, 18, 20–22]. The prognostic implications of BCL6 for PCNS-DLBCL are largely conflicting [10, 16, 19–25]. The results of this study further suggest that BCL6 may have little, if any, prognostic value for PCNS-DLBCL. Non-GCB phenotype tumors were the predominant PCNS-DLBCL type in this study, and no association between cell of origin and prognosis was found, which is consistent with previous reports [9, 26, 27]. Unexpectedly, patients with high serum LDH levels had a more favorable PFS than that of patients with normal serum LDH levels. In addition, the results of this study show that alleged prognostic scoring systems, including Nottingham-Barcelona and MSKCC, reflect PFS but not OS. The reason for these discrepancies is not known, and we were unable to deduce the reasons over the course of this study; however, these results might support the idea that PCNS-DLBCL prognosis is dependent on multiple, complex factors. This study has some limitations. It was a retrospective study, and therapeutic modalities were not completely homogeneous between patients. However, to the best of our knowledge, this study is the largest performed on the expression of MYC, BCL2 and BCL6 PCNS-DLBCL with long-term follow up, and the first report on the relationship of these factors to clinicopathological features in Asian patients.

## Conclusions

This study demonstrated that the expression of MYC and BCL2 may be of prognostic value in patients with PCNS-DLBCL when combined with existing prognostic tools and factors.

## Abbreviations

CHOD/BVAM, cyclophosphamide, doxorubicin, vincristine and dexamethasone/bis-chloronitrosourea, cytosine arabinoside and methotrexate; CHOP, cyclophosphamide, doxorubicin, vincristine and prednisone; CI, confidence interval; CNS, central nervous system; COPADM, cyclophosphamide, vincristine, prednisolone, doxorubicin and methotrexate; CSF, cerebrospinal fluid; DLBCL, diffuse large B-cell lymphoma; ECOG PS, The Eastern Cooperative Oncology Group performance score; GCB, germinal center B-cell-like; GTR, grossly total resection; HD-MTX, high-dose methotrexate; HR, hazard ratio; IELSG, The international extranodal lymphoma study group; IHC, immunohistochemistry; IPI, international prognostic index; IT-MTX, intrathecal methotrexate; KPS, Karnofsky Performance Status score; LDH, lactate dehydrogenase; MSKCC, Memorial Sloan-Kettering Cancer Center; MVP, combined chemotherapy regimen of high-dose methotrexate, vincristine and procarbazine; N-B, Nottingham- Barcelona; OS, overall survival; PCNS-DLBCL, primary diffuse large B-cell lymphoma of the central nervous system; PFS, progression-free survival; RT, radiotherapy; STR, subtotal resection; WHO, World Health Organization; yr, year

## Additional files

Additional file 1: Figure S1. Correlation of MYC, BCL2 and BCL6 IHC score. Correlations between MYC and BCL2 (left upper), MYC and BCL6 (right upper), and BCL2 and BCL6 (lower) IHC score was compared using Spearman correlation test. (PPT 343 kb) Additional file 2:
**Table S1.** Correlation of BCL6 expression and clinicopathological variables; **Table S2.** MYC translocation and copy number change in MYC positive cases. (DOCX 24 kb) Additional file 3: Figure S2. Progression-free survival (PFS) and overall survival (OS) of patients with PCNS-DLBCL treated with MVP-RT according to MYC or BCL2 expression status. (A and B) PFS and OS according to MYC protein expression status (cutoff score 40) are plotted using the Kaplan-Meier method and analyzed by the log-rank test. PFS and OS according to BCL2 protein expression status using a cutoff score of 30 (C and D), or with a cutoff score of 60 (E and F) are plotted using the Kaplan-Meier method and analyzed by the log-rank test. (TIF 12287 kb) Additional file 4: Dataset of 114 patients with PCNS-DLBCL. (XLSX 56 kb)
