# Supplementary material for: MYC and BCL2 overexpression is associated with a higher class of Memorial Sloan-Kettering Cancer Center prognostic model and poor clinical outcome in primary diffuse large B-cell lymphoma of the central nervous system
Source: BMC Cancer. 2016 Jun 10;16:363. doi: 10.1186/s12885-016-2397-8 (PMC4903010; doi:10.1186/s12885-016-2397-8)
Supplement: Additional file 1: Figure S1. — Correlation of MYC, BCL2 and BCL6 IHC score. Correlations between MYC and BCL2 (left upper), MYC and BCL6 (right upper), and BCL2 and BCL6 (lower) IHC score was compared using Spearman correlation test. (PPT 343 kb) [file 12885_2016_2397_MOESM1_ESM.ppt]

## Slide 1
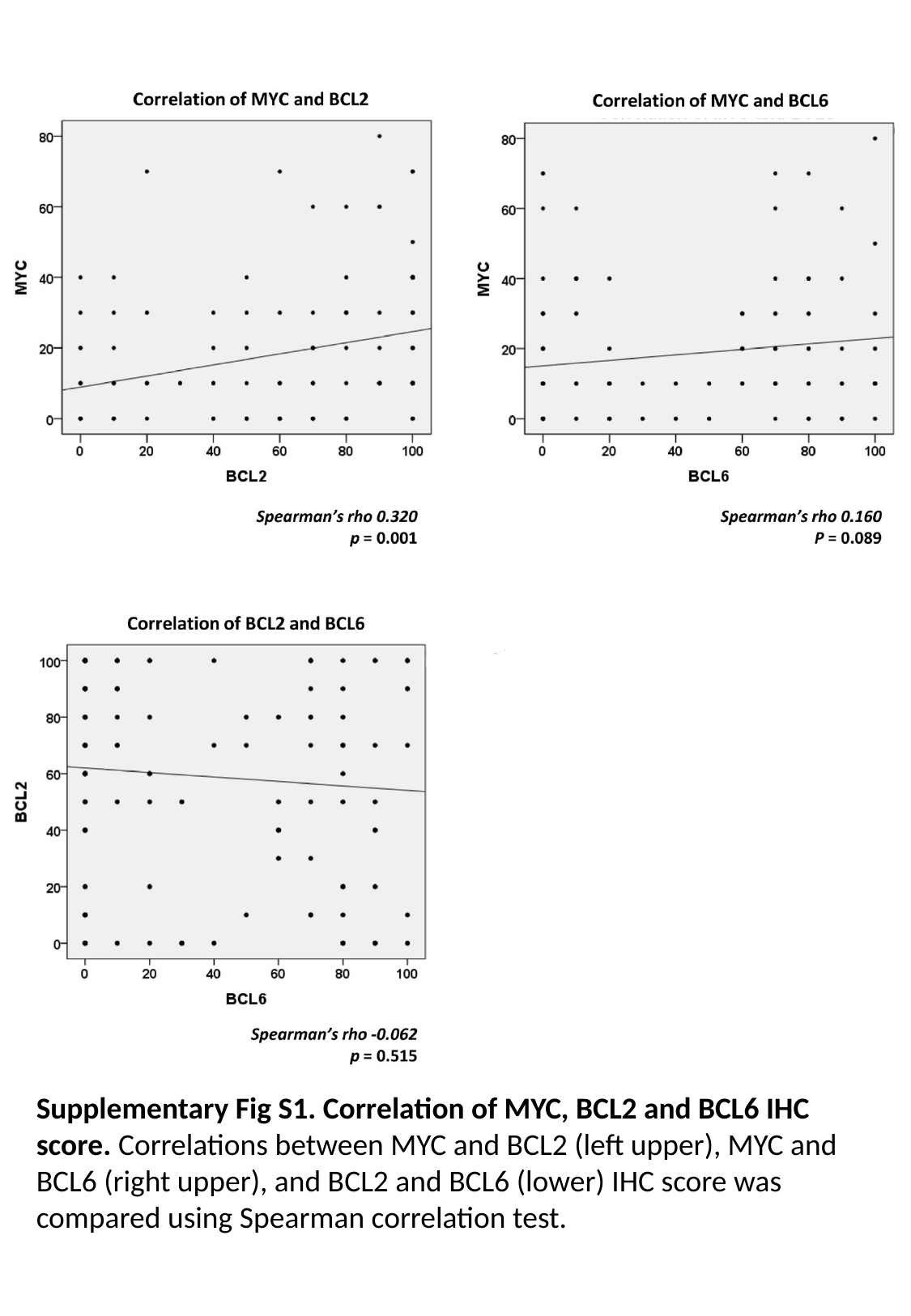

Supplementary Fig S1. Correlation of MYC, BCL2 and BCL6 IHC score. Correlations between MYC and BCL2 (left upper), MYC and BCL6 (right upper), and BCL2 and BCL6 (lower) IHC score was compared using Spearman correlation test.
