# Supplementary material for: MYC and BCL2 overexpression is associated with a higher class of Memorial Sloan-Kettering Cancer Center prognostic model and poor clinical outcome in primary diffuse large B-cell lymphoma of the central nervous system
Source: BMC Cancer. 2016 Jun 10;16:363. doi: 10.1186/s12885-016-2397-8 (PMC4903010; doi:10.1186/s12885-016-2397-8)
Supplement: Additional file 2: — Table S1. Correlation of BCL6 expression and clinicopathological variables; Table S2. MYC translocation and copy number change in MYC positive cases. (DOCX 24 kb) [file 12885_2016_2397_MOESM2_ESM.docx]

**Table S1. Correlation of BCL6 expression and clinicopathological variables**

|  |  | BCL6, n (%) | | |
| --- | --- | --- | --- | --- |
| Variables^a^ |  | <50% | ≥50% | *P* |
| Age (yr) | mean ±SD | 57.8±13 | 59.7±14.7 | 0.462 |
| Age (yr) | ≤50  >50 | 13 (56.5)  50 (54.9) | 10 (43.5)  41 (45.1) | 0.892 |
| Age (yr) | ≤60  >60 | 33 (60.0)  30 (50.8) | 22 (40.0)  29 (49.2) | 0.326 |
| Sex | M  F | 39 (60.0  24 (49.0) | 26 (40.0)  25 (51.0) | 0.241 |
| ECOG PS | 0, 1  2-4 | 41 (56.2)  21 (53.8) | 32 (43.8)  18 (46.2) | 0.814 |
| KPS | ≥70  <70 | 52 (53.6)  10 (71.4) | 45 (46.4)  4 (28.6) | 0.258^‡^ |
| B symptoms | Absent  Present | 60 (55.0)  3 (60.0) | 49 (45.0)  2 (40.0) | >0.999^‡^ |
| Serum LDH | Normal  Elevated | 36 (52.9)  21 (53.8) | 32 (47.1)  18 (46.2) | 0.928 |
| Cell of origin | GCB  Non-GCB | 8 (33.3)  55 (61.8) | 16 (66.7)  34 (38.2) | 0.013 |
| Involvement of deep structure | Absent  Present | 12 (40.0)  51 (60.7) | 18 (60.0)  33 (39.3) | 0.050 |
| Extent of disease | Unifocal  Multifocal | 19 (45.2)  44 (61.1) | 23 (54.8)  28 (38.9) | 0.100 |
| Ocular involvement | Absent  Present | 49 (55.1)  14 (56.0) | 40 (44.9)  11 (44.0) | 0.933 |
| CSF protein | Normal  Elevated | 24 (61.5)  24 (45.3) | 15 (38.5)  29 (54.7) | 0.123 |
| CSF cytology | Negative  Positive | 45 (54.9)  5 (35.7) | 37 (45.1)  9 (64.3) | 0.250^‡^ |
| IELSG | 0-2  3-5 | 22 (51.2)  26 (54.2) | 21 (48.8)  22 (45.8) | 0.774 |
| Nottingham – Barcelona | 0-1  2-3 | 26 (49.1)  36 (61.0) | 27 (50.9)  23 (39.0) | 0.204 |
| MSKCC class | 1  2-3 | 12 (57.1)  50 (54.9) | 9 (42.9)  41 (45.1) | 0.855 |

Abbreviations: ECOG PS, The Eastern Cooperative Oncology Group performance score; KPS, Karnofsky performance status score; LDH, lactate dehydrogenase; GCB, germinal center B cell-like; CSF, cerebrospinal fluid; IELSG, the International Extranodal Lymphoma Study Group; MSKCC, Memorial Sloan Kettering Cancer Center.

^a^Some cases have missing values that lacked the information about the variables.

^b^Fisher’s exact test

**Table S2. MYC translocation and copy number change in MYC positive cases**

| Case No. | MYC IHC score | MYC translocation | Gene Copy number |
| --- | --- | --- | --- |
| Case 1 | 60 | fail | <2 |
| Case 2 | 60 | fail | 3.5 |
| Case 3 | 40 | negative | <2 |
| Case 4 | 70 | negative | <2 |
| Case 5 | 70 | negative | <2 |
| Case 6 | 40 | negative | <2 |
| Case 7 | 50 | negative | <2 |
| Case 8 | 40 | negative | <2 |
| Case 9 | 40 | positive | - |
| Case 10 | 40 | positive | - |
| Case 11 | 60 | negative | <2 |
| Case 12 | 60 | negative | <2 |
| Case 13 | 80 | negative | <2 |
| Case 14 | 40 | negative | <2 |
| Case 15 | 40 | negative | <2 |
| Case 16 | 40 | fail | <2 |
| Case 17 | 40 | negative | 2.2 |
| Case 18 | 40 | negative | <2 |
| Case 19 | 70 | negative | <2 |
| Case 20 | 70 | negative | 3.2 |

Abbreviation: IHC, immunohistochemistry
