# Supplementary figures and images for: MYC and BCL2 overexpression is associated with a higher class of Memorial Sloan-Kettering Cancer Center prognostic model and poor clinical outcome in primary diffuse large B-cell lymphoma of the central nervous system
Source: BMC Cancer. 2016 Jun 10;16:363. doi: 10.1186/s12885-016-2397-8 (PMC4903010; doi:10.1186/s12885-016-2397-8)

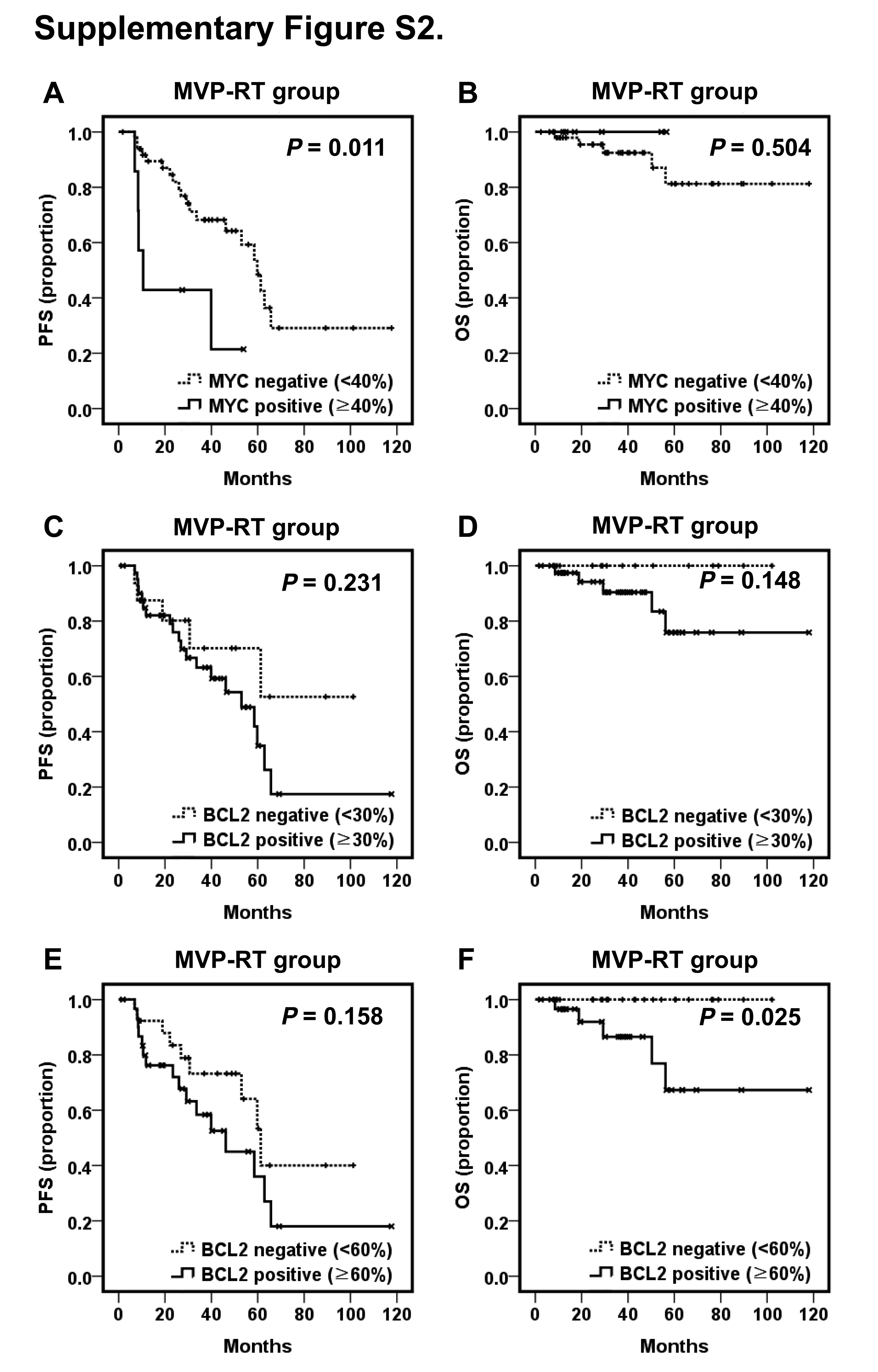

Supplement: Additional file 3: Figure S2. — Progression-free survival (PFS) and overall survival (OS) of patients with PCNS-DLBCL treated with MVP-RT according to MYC or BCL2 expression status. (A and B) PFS and OS according to MYC protein expression status (cutoff score 40) are plotted using the Kaplan-Meier method and analyzed by the log-rank test. PFS and OS according to BCL2 protein expression status using a cutoff score of 30 (C and D), or with a cutoff score of 60 (E and F) are plotted using the Kaplan-Meier method and analyzed by the log-rank test. (TIF 12287 kb) [file 12885_2016_2397_MOESM3_ESM.tif]
